# Optical coherence tomography artefact burden predicts cognitive decline and incident dementia

**DOI:** 10.3389/fnagi.2026.1802753

**Published:** 2026-05-15

**Authors:** Serene Jia Ning Loke, Reuben Jyong Kiat Foo, Nur Fidyana Binte Abdul Gani, Damon Wong, Bingyao Tan, An Qi Toh, Narayanaswamy Venketasubramanian, Boon Yeow Tan, Christopher Li-Hsian Chen, Leopold Schmetterer, Jacqueline Chua

**Affiliations:** 1 Singapore Eye Research Institute, Singapore National Eye Centre, Singapore, Singapore; 2 Yong Loo Lin School of Medicine, National University of Singapore, Singapore, Singapore; 3 SERI-NTU Advanced Ocular Engineering (STANCE), Singapore, Singapore; 4 School of Chemical and Biological Engineering, Nanyang Technological University, Singapore, Singapore; 5 Ophthalmology and Visual Sciences Academic Clinical Program, Duke-NUS Medical School, National University of Singapore, Singapore, Singapore; 6 Departments of Pharmacology and Psychological Medicine, Memory Aging and Cognition Centre, Yong Loo Lin School of Medicine, National University of Singapore, Singapore, Singapore; 7 Raffles Neuroscience Centre, Raffles Hospital, Singapore, Singapore; 8 St Luke’s Hospital, Singapore, Singapore; 9 Department of Clinical Pharmacology, Medical University Vienna, Wien, Austria; 10 Aier Eye Hospital Group, Changsha, China; 11 Fondation Ophtalmologique Adolphe De Rothschild, Paris, France

**Keywords:** Alzheimer’s disease, cognitive decline, cognitive impairment, dementia, imaging artefacts, neurodegeneration, optical coherence tomography (OCT), scan quality

## Abstract

**Introduction:**

Scalable, low-burden biomarkers for predicting cognitive decline remain needed. We hypothesised that OCT acquisition irregularities (commonly termed scan artefacts), which may reflect visuomotor and attentional instability during imaging, carry prognostic information for cognitive outcomes.

**Methods:**

We studied 507 Asian memory clinic participants who underwent baseline OCT imaging and comprehensive neuropsychological assessment annually for up to 5 years. OCT artefacts (motion, shadow, off-centre, refractive shift, out-of-boundary, and tilt) and signal strength were graded at baseline. Clinically significant cognitive decline was defined as a ≥ 0.5 standard deviation decrease in global cognitive z-score from baseline at any follow-up visit. Cox proportional hazards models evaluated associations between artefact burden and incident cognitive decline, adjusting for demographics, education, diabetes, and baseline cognitive status/diagnosis. Among participants with cognitive impairment no dementia (CIND) at baseline (*n* = 222), we additionally assessed conversion to dementia.

**Results:**

Cognitive decline occurred in 289 participants (57%). Presence of any artefact was associated with higher risk of decline (HR = 1.37, 95% CI = 1.12–1.68, *p* = 0.003). Among 222 participants with baseline CIND, bilateral artefacts were associated with higher risk of conversion to dementia (HR = 1.82, 95% CI = 1.10–3.02, *p* = 0.019).

**Conclusion:**

Routine OCT acquisition irregularities may provide an opportunistic, low-burden prognostic signal for cognitive vulnerability and dementia progression, complementing established neurodegenerative biomarkers.

## 1 Introduction

Dementia is a major public health challenge, with approximately 10 million new cases diagnosed globally each year (GBD 2016 Dementia Collaborators, 2019). It not only impairs quality of life but also imposes substantial socioeconomic burdens on patients, families, and healthcare systems. In the absence of effective curative therapies, reducing the impact of dementia relies heavily on prevention efforts and on detecting cases early enough to initiate supportive interventions (Livingston et al., 2024).

Conventional tools such as the Mini-Mental State Examination (MMSE), Montreal Cognitive Assessment (MoCA), and comprehensive neuropsychological batteries remain the gold standard for assessing cognitive status (Weinstein et al., 2022). However, these tools are time-consuming, influenced by education and cultural factors, and may fail to detect subtle changes in early disease stages (Trenkle et al., 2007). This has driven increasing interest in objective, non-invasive biomarkers that can complement clinical evaluation and improve risk stratification.

Optical coherence tomography (OCT) offers high-resolution imaging of the retina, a structure that shares embryological and physiological features with the brain (Gaire et al., 2024). Prior studies have shown that retinal thinning, particularly in the ganglion cell–inner plexiform layer (GCIPL), may predict future cognitive decline (Eppenberger et al., 2024). Yet, the interpretation of OCT scans is often hampered by poor image quality. Recent cross-sectional studies, including our recent work, have demonstrated that poor-quality scans are more common in dementia compared with cognitively normal individuals (Abraham et al., 2021; Foo et al., 2025). These artefacts, such as motion, or off-center alignment, are typically dismissed as technical limitations or poor scan quality to be excluded from analyses. However, their increased prevalence in cognitively impaired populations may reflect behavioural or neurological factors such as impaired fixation, reduced attention, or difficulty following instructions, raising the possibility that they contain clinically meaningful information.

To date, no longitudinal study has systematically evaluated whether OCT artefacts themselves predict cognitive outcomes. In this study, we analysed a large, well-characterised Asian memory clinic cohort with up to five years of follow-up. We investigated whether baseline OCT artefacts were associated with risk of subsequent cognitive decline and with conversion from cognitive impairment no dementia (CIND) to dementia. Because artefacts are routinely graded during OCT quality control, they are essentially a “free” by-product of clinical imaging, and may offer a novel, easily obtainable biomarker of neurodegenerative risk.

## 2 Materials and methods

### 2.1 Study participants

The Memory Aging and Cognition Centre (MACC) Harmonisation cohort is a longitudinal memory clinic–based study conducted at two sites in Singapore: The National University Hospital and St. Luke’s Hospital. Participants were followed for up to five years. Eligible individuals were aged ≥ 50 years and had sufficient language proficiency to complete standardised neuropsychological assessments. Participants were excluded if they had clinically relevant conditions that would significantly impede cognitive assessment, including substance use disorder, major psychiatric illness, brain tumours, multiple sclerosis, epilepsy, traumatic brain injury resulting in cognitive impairment, or self-reported diagnosis of glaucoma due to the risk of acute angle-closure glaucoma with mydriatic eyedrops.

For the present analysis, participants were required to have undergone baseline retinal OCT imaging and completed comprehensive neuropsychological testing at baseline and during each of the five annual follow-up visits.

### 2.2 Standard protocol approvals, registrations, and patient consents

The study was approved by the National Healthcare Group Domain-Specific Review Board (DSRB Ref: 2010/00017 and 2018/01098) and conducted in accordance with the Declaration of Helsinki and its subsequent amendments. Written informed consent was obtained from all participants or their legal representatives prior to enrolment.

### 2.3 Participant characteristics and clinical factors

Information on age, sex, and education was collected using standardised interviewer-administered questionnaires. Histories of systemic diseases such as diabetes mellitus and hypertension were obtained from participants and verified against their medical records.

### 2.4 Neurocognitive assessment and diagnoses

Neurocognitive diagnoses were determined at regular consensus meetings involving study clinicians and neuropsychologists. Annual cognitive assessments included the Mini-Mental State Examination (MMSE), and a locally validated neuropsychological battery, administered by trained research psychologists in the participants’ native language (English, Mandarin, or Malay), as previously described (Xu et al., 2015). Participants without objective impairment were classified as having no cognitive impairment (NCI). Cognitive impairment no dementia (CIND) was defined as impairment in one or more cognitive domains, indicated by performance at least 1.5 standard deviations below education-adjusted cutoff values on any test, without evidence of loss of independent daily functioning. Dementia was diagnosed according to criteria from the Diagnostic and Statistical Manual of Mental Disorders, Fourth Edition (DSM-IV) (Hilal et al., 2015).

### 2.5 Outcome definition

The primary outcome was clinically significant cognitive decline over five years, defined as a binary variable (decline vs. no decline). Cognitive performance was assessed across six domains: attention, executive function, language, visuomotor speed, visuospatial function, and memory. Up to 14 tests from the validated Neurological Disorders and Stroke–Canadian Stroke Network (NINDS-CSN) battery was administered at baseline and annually (Phua et al., 2018).

For each participant, a standardised global z-score was calculated at baseline and follow-up visits, reflecting performance relative to the mean and standard deviation (SD) of the NCI reference group. Significant cognitive decline was defined as a decrease of ≥ 0.5 SD in the global z-score from baseline at any follow-up year (Hessen et al., 2017; Phua et al., 2018), a threshold commonly used to indicate clinically meaningful decline with potential impact on daily functioning, supported by longitudinal evidence showing that older adults with rapid cognitive decline exhibited an average decrease of approximately 0.5 SD in composite cognitive z-scores (Wolinsky et al., 2006). The secondary outcome was progression from CIND to dementia, defined as conversion to dementia during follow-up (progressors vs. non-progressors).

Of these 581 participants, 74 did not have any follow-up neuropsychological assessments. This resulted in a final sample size of 507 participants for the cognitive decline analysis. Due to the low risk of incident dementia in NCI participants, we focused on CIND participants in analysing incident dementia over the follow-up period. Among the 507 participants, 222 were classified as CIND; this was the final sample size for the incident dementia analysis (Supplementary Figure 1).

### 2.6 Ocular assessments

All participants also underwent auto-refraction–keratometry using the Canon RK-5 Autorefractor Keratometer (Canon Inc., Tokyo, Japan), with spherical equivalent calculated as the spherical value plus half the cylindrical value (diopters). Fundus imaging was conducted with a non-mydriatic digital retinal camera (Canon CR-1 Mark II, Canon Inc., Ota, Tokyo, Japan) following pupil dilation with 1% tropicamide. Fundus photographs were then graded by a trained assessor, masked to participant characteristics, for ocular pathologies such as age-related macular degeneration, diabetic retinopathy, glaucoma, maculopathy, cataracts, and other retinopathies (Chua et al., 2022b).

OCT imaging was performed using the Cirrus 5000 spectral domain OCT system (Carl Zeiss Meditec, Dublin, CA, United States). Each eye was scanned with a 200 A-scan × 200 B-scan raster protocol (6 × 6 mm^2^) centered on the optic disc, with FastTrac™ motion correction enabled to minimise artefacts from eye movement and blinking. All technicians received rigorous training and standardised procedures in place to ensure consistent, high-quality scans (Chua et al., 2022a; Chua et al., 2025); when scan quality was deemed suboptimal (e.g., due to poor centration, motion, or low signal strength), repeat acquisitions were performed at the technician’s discretion to obtain the best achievable image. Subsequent quality grading and artefact classification were conducted using predefined criteria as described below.

Peripapillary retinal nerve fibre layer (RNFL) thickness obtained from optic nerve head scans is the most widely studied OCT metric in Alzheimer’s disease and mild cognitive impairment, as confirmed by recent meta-analyses (den Haan et al., 2017; Chan et al., 2019). Scan signal strength and peripapillary retinal nerve fibre layer (RNFL) thickness measurements were taken from the Cirrus Review Software (software version 11.0.0.29946).

### 2.7 Definition of participant-level artefact variables

The procedures for scan quality assessment and grading criteria for artefact grading have been described previously (Foo et al., 2025). In brief, OCT scans were manually reviewed by trained graders blinded to participant characteristics and cognitive diagnoses, for the presence of specific artefact types, including motion (horizontal discontinuity of retinal vessels due to participant’s eye movements), shadows (localised signal loss or light blockage from ocular opacities), off-center (scan not centered on the optic disc), refractive shifts (alteration in intensity between adjacent scans due to blinking or changes in corneal surface refractive index), out of boundary (B-scans are cut off at the top or bottom such that some or all retinal layers are not fully imaged), and tilt (poor alignment causing part of the image to be low-signal or out of focus) (Supplementary Figure 2).

Artefact grading was performed manually using predefined criteria by trained graders (Chua et al., 2022a; Chua et al., 2025). Reliability was high, with intra-grader weighted κ values ranging from 0.87 to 0.92 and inter-grader weighted κ values ranging from 0.79 to 0.89 across artefact categories, indicating good to almost perfect agreement.

Importantly, overall scan quality was evaluated separately from artefact presence. Good-quality scans were defined as those that were well centered, with the optic disc in clear focus, retinal layers well visualised, and signal strength > 6, even if minor artefacts were present outside the region of interest. Poor-quality scans were defined as those failing these criteria (e.g., signal strength ≤ 5, severe motion artefacts, inconsistent signal intensity, or segmentation failure causing poor contrast in the en face image). Thus, a scan could contain one or more artefacts yet still be classified as good quality if it retained adequate centering, focus, layer definition, and signal strength.

For analysis, each artefact type (motion, shadow, low signal strength ≤ 5, off-center, refractive shift, out-of-boundary, and tilt) and scan quality were coded as binary variables (present in either eye vs. absent). Two composite artefact variables were created: (1) artefacts in any eye (at least one artefact in either eye) and (2) artefacts in both eyes ( ≥ 1 artefact present bilaterally). Bilateral artefacts were considered a more stringent marker of generalised imaging difficulty and may be more consistent with participant-level attentional or visuomotor limitations, whereas unilateral artefacts may be more susceptible to confounding by eye-specific or scan-specific factors. To further evaluate the incremental value of OCT artefacts in a clinical logistic regression model, we also combined all 10 artefact variables into a composite model (i.e., seven artefact types, scan quality, and two composite variables). For clarity, the term “artefact burden” in this study refers to categorical indicators of artefact presence and laterality, rather than a continuous or dose-dependent measure.

### 2.8 Statistical analysis

Statistical analysis was performed using STATA (StataCorp. 2023. Release 16.1. College Station, TX: Statacorp LLC. All statistical tests were conducted at a significance level of 0.05. Participant characteristics were compared between participants who developed cognitive decline and those who did not over five years. Normality of continuous variables was assessed using the Shapiro–Wilk test. As all variables deviated from a normal distribution, continuous variables were summarised as median with interquartile range (IQR) and compared using the Kruskal–Wallis test. Categorical variables were summarised as counts (percentages) and compared using chi-squared tests or Fisher’s exact tests, when appropriate. For eye-level analyses, generalised estimating equations (GEE) were used to account for within-subject correlation between eyes. Linear regression with GEE, adjusted for age and sex, was applied to compare continuous variables between groups.

Time-to-event analyses were performed to evaluate the association between OCT artefacts and the risk of cognitive decline and dementia conversion. For cognitive decline, participants were followed from baseline until the first visit at which decline was observed or until their last available follow-up. For dementia conversion, analyses were restricted to participants with CIND at baseline and followed until conversion to dementia or censoring. Cox proportional hazards regression models were used to estimate hazard ratios (HRs) with 95% confidence intervals (CIs). Models were adjusted for age, sex, education, diabetes mellitus, and baseline diagnosis, as appropriate. Robust standard errors clustered by participant ID accounted for within-subject correlation. Kaplan–Meier curves were generated to illustrate the cumulative incidence of outcomes stratified by artefact status.

## 3 Results

Of 581 participants with baseline OCT imaging, 74 were excluded due to a lack of follow-up neuropsychological assessments. A total of 507 participants were included in the cognitive decline analysis, of whom 289 (57%) developed cognitive decline and 218 (43%) remained stable without measurable decline over 5 years. Participant flow across the study is further illustrated in Supplementary Figure 2.

### 3.1 Demographic and clinical characteristics

Baseline characteristics by cognitive outcome are shown in Table 1. Participants who developed cognitive decline were significantly older [median (IQR) 75 (9) years] compared with those without decline [72 (11) years; *P* < 0.001] and had fewer years of education (6 (7) vs. 10 (7); *P* < 0.001). In terms of baseline diagnosis, participants with cognitive decline were more likely to have CIND (41% vs. 47%) or dementia (49% vs. 15%), while those without decline were more often cognitively normal (38% vs. 10%, *P* < 0.001). Diabetes mellitus was more prevalent in the decline group (37% vs. 28%, *P* = 0.030). Baseline cognitive performance was also poorer among participants who declined, with lower MMSE scores (21 (9) vs. 26 (5), *P* < 0.001). No significant differences were observed between groups in terms of sex, hypertension, or blood pressure (all *P* > 0.120).

**TABLE 1:** Comparison of demographic and clinical characteristics between subjects with and without cognitive decline over a 5-year follow-up period.

Characteristics	With cognitive decline	Without cognitive decline	*P*-value
Number of subjects	289	218	
Age (years)	75 (9)	72 (11)	**< 0.001**
Sex, male	133 (46%)	89 (41%)	0.243
Education (years)	6 (7)	10 (7)	**< 0.001**
Baseline diagnosis			**< 0.001**
No cognitive impairment	29 (10%)	82 (38%)	
Cognitive impairment, no dementia	119 (41%)	103 (47%)	
Dementia	141 (49%)	33 (15%)	
Diabetes mellitus, yes	106 (37%)	60 (28%)	**0.030**
Hypertension, yes	203 (71%)	140 (64%)	0.120
Blood pressure			
Systolic blood pressure (mmHg)	141 (26)	140 (22)	0.421
Diastolic blood pressure (mmHg)	73 (14)	73 (12)	0.639
Cognitive tests			
MMSE total score	21 (9)	26 (5)	**< 0.001**
Ocular characteristics			
Number of eyes	547	425	
Patients with both eyes included	258 (89%)	207 (95%)	
Patients with either eye included	31 (11%)	11 (5%)	
Spherical equivalent (diopters) *	-0.50 (2)	-0.38 (2)	0.898
Signal strength of scan (0 poor to 10 good) *	8 (1)	8 (2)	0.798
RNFL thickness (μm) *	87 (20)	90 (15)	0.070
Ocular disease in any eye	101 (39%)	71 (34%)	0.207
Scan quality			
Good scan quality in either eye	235 (81%)	192 (88%)	**0.039**
Poor scan quality in both eyes	54 (19%)	26 (12%)	
Artefacts			
Motion	165 (57%)	95 (44%)	**0.003**
Shadows	152 (53%)	98 (45%)	0.088
Low signal strength ( ≤ 5)	29 (10%)	24 (11%)	0.723
Off-center	39 (13%)	14 (6%)	**0.010**
Refractive shift	27 (9%)	12 (6%)	0.108
Out of boundary	32 (11%)	16 (7%)	0.155
Tilt	7 (2%)	7 (3%)	0.592
Artefacts in any eye	232 (80%)	143 (66%)	**< 0.001**
Artefacts in both eyes	115 (40%)	71 (33%)	0.095

Data provided in median (IQR) or number (%). MMSE, Mini-Mental State Examination. *P*-values were obtained using Kruskal-Wallis test for continuous variables and the chi-squared test for categorical variables; Fisher’s exact test was applied when expected cell counts were < 5. *P-values were obtained from generalised estimating equations (GEE) accounting for within-subject correlation between eyes, adjusted for age and sex. Bold values denote statistical significance at *p* < 0.05.

### 3.2 Ocular characteristics

There were no significant differences in spherical equivalent refractive error (-0.50 (2) vs. -0.38 (2) diopters, *P* = 0.898), scan signal strength (8 (1) vs. 8 (2), *P* = 0.798), RNFL thickness (87 (20) μm vs. 90 (15) μm, *P* = 0.070), or presence of ocular disease (39% vs. 34%, *P* = 0.207) between groups.

Poor scan quality in both eyes was more frequent in the decline group (19% vs. 12%, *P* = 0.039). Motion artefacts (57% vs. 44%, *P* = 0.003) and off-center artefacts (13% vs. 6%, *P* = 0.010) were also more common among those with decline, while other artefact types were not (all *P* > 0.088). Overall, the presence of any artefact was significantly higher in participants with decline (80% vs. 66%, *P* < 0.001).

Artefacts, particularly poor scan quality, motion, and off-center artefacts (*P* ≤ 0.030), were significantly more frequent in participants with dementia compared with those with CIND or NCI (Supplementary Table 1).

### 3.3 Associations between OCT artefacts and risk of cognitive decline

**FIGURE 1:**
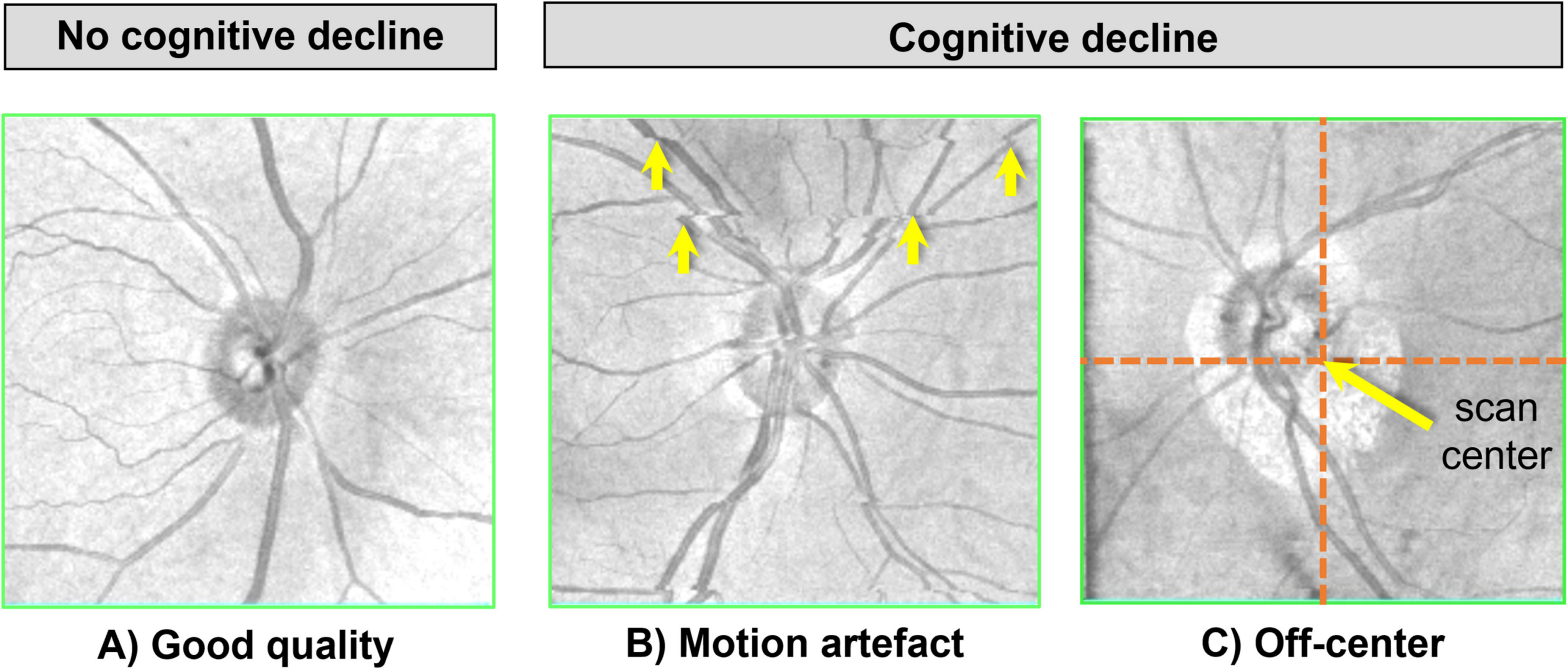
Representative OCT scans illustrating artefact patterns. **(A)** High-quality scan without visible artefacts from a participant without cognitive decline. In contrast, representative scans from participants with cognitive decline demonstrate **(B)** motion artefact, characterised by horizontal discontinuities of retinal vessels (yellow arrows), and **(C)** off-center artefact, indicated by displacement of the optic disc relative to the scan center (orange dashed lines and arrow).

As shown in the age-adjusted analyses (Table 2), poor-quality scan (HR 1.43, 95% CI 1.15–1.79, *P* = 0.002), motion artefact (HR 1.35, 95% CI 1.11–1.63, *P* = 0.002), off-center (HR 1.42, 95% CI 1.10–1.83, *P* = 0.006), and the presence of any artefact (HR 1.50, 95% CI 1.19–1.90, *P* = 0.001) were associated with increased risk of cognitive decline. After further adjustment for sex, education, diabetes mellitus, and baseline diagnosis, only the presence of any artefact remained significantly associated with risk of cognitive decline, with participants showing a 37% higher risk (HR 1.37, 95% CI 1.12–1.68, *P* = 0.003). After additional adjustment for the presence of ocular disease, this association remained statistically significant, with participants demonstrating a 34% higher risk of cognitive decline (HR 1.38, 95% CI 1.06–1.79, P = 0.017) in the fully adjusted model (Supplementary Table 2). These findings suggest that the relationship between OCT artefacts and cognitive decline is independent of ocular pathology. In terms of predictive performance, the multivariable logistic regression model showed that using a composite of all artefact variables provided significant improvement in predicting risk of cognitive decline (AUC = 0.693, 95% CI = 0.646–0.740, *P* = 0.023), compared to baseline clinical variables (Table 3). Figure 1 illustrates representative examples of good quality, motion, and off-center scans in cognitively stable versus declining participants.

**TABLE 2:** Associations between optical coherence tomography artefacts at baseline and risk of cognitive decline (*n* = 507).

OCT artefact parameter	Model 1 HR (95% CI)	Model 1 *P*-value	Model 2 HR (95% CI)	Model 2 *P*-value
Poor scan quality	**1.43 (1.15–1.79)**	**0.002**	1.07 (0.87–1.32)	0.511
Types of artefacts				
Motion	**1.35 (1.11–1.63)**	**0.002**	1.16 (0.97–1.38)	0.106
Shadows	1.14 (0.95–1.38)	0.166	–	–
Low signal strength (≤5)	0.98 (0.75–1.28)	0.889	–	–
Off-center	**1.42 (1.10–1.83)**	**0.006**	1.16 (0.89–1.51)	0.262
Refractive shift	1.29 (0.93–1.80)	0.126	**–**	**–**
Out of boundary	1.18 (0.85–1.64)	0.319	**–**	**–**
Tilt	0.92 (0.47–1.79)	0.796	**–**	**–**
Presence of artefacts				
Artefacts in any eye	**1.50 (1.19–1.90)**	**0.001**	**1.37 (1.12–1.68)**	**0.003**
Artefacts in both eyes	1.20 (0.99–1.45)	0.052	–	–

CI, confidence interval; HR, hazard ratio. Artefacts in any eye = presence of ≥ 1 artefact in either eye. Artefacts in both eyes = presence of ≥ 1 artefact in each eye. *P*-values were obtained from Cox proportional hazards regression models. Model 1—Adjusted for age. Model 2—Adjusted for age, sex, education, diabetes mellitus, and baseline diagnosis. Bold values denote statistical significance at *p* < 0.05.

**TABLE 3:** Discrimination performance of baseline and exposure-augmented logistic regression models for prediction of cognitive decline, as well as conversion from cognitive impairment no dementia (CIND) to incident dementia.

	Cognitive decline (*n* = 507)				Incident dementia (*n* = 222)			
	Model 1		Model 2		Model 1		Model 2	
Subjects	AUC (95% CI)	*P*-value	AUC (95% CI)	*P*-value	AUC (95% CI)	*P*-value	AUC (95% CI)	*P*-value
Baseline	0.622 (0.573–0.671)	–	0.659 (0.610–0.707)	–	0.619 (0.540–0.699)	–	0.644 (0.565–0.722)	–
All artefact variables	**0.674 (0.627–0.722)**	**0.008**	**0.693 (0.646–0.740)**	**0.023**	0.678 (0.597–0.759)	0.100	0.692 (0.610–0.775)	0.150

AUC, area under curve; CI, confidence interval. All artefact variables include poor scan quality, the seven artefact types, and two composite artefact variables. Artefacts in any eye = presence of ≥ 1 artefact in either eye. Artefacts in both eyes = presence of ≥ 1 artefact in each eye. *P*-values were obtained from DeLong’s test. Model 1—Adjusted for age. Model 2—Adjusted for age, sex, education, and diabetes mellitus. Bold values denote statistical significance at *p* < 0.05.

### 3.4 Associations between OCT artefacts and incident dementia

**FIGURE 2:**
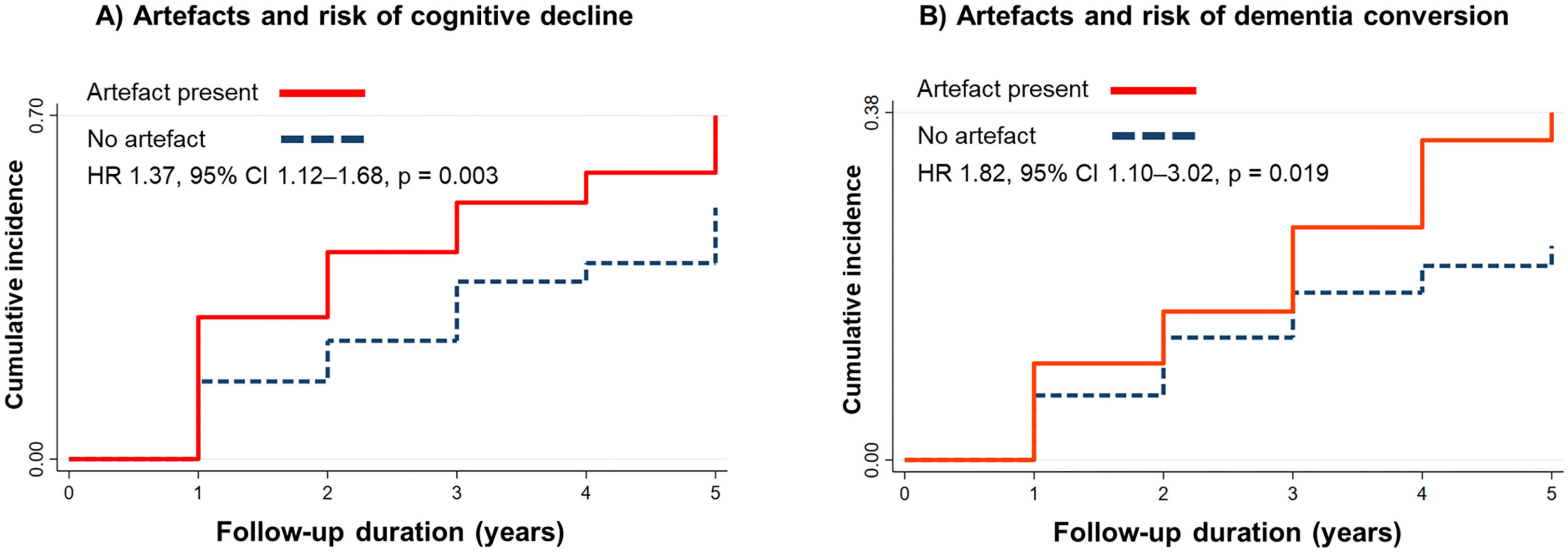
Kaplan–Meier curves for cognitive outcomes according to OCT artefact status. **(A)** Participants with artefacts in either eye showed a higher cumulative incidence of cognitive decline. **(B)** Participants with artefacts in both eyes had an increased risk of conversion from cognitive impairment no dementia (CIND) to dementia.

Among 222 participants with CIND at baseline, 58 (26%) converted to dementia over follow-up, while 164 (74%) subjects did not. Participants who progressed were significantly older than non-progressors (*P* = 0.007; Supplementary Table 3). As shown in Table 4, the presence of artefacts in both eyes was significantly associated with increased risk of conversion from CIND to dementia. In the fully adjusted model (age, sex, education, and diabetes mellitus), participants with artefacts in both eyes had an 82% higher risk of dementia conversion (HR 1.82, 95% CI 1.10–3.02, *P* = 0.019). After further adjustment for ocular disease, the association remained significant, with a 70% higher risk of dementia conversion (HR 1.70, 95% CI 1.02–2.83, P = 0.040) in the fully adjusted model (Supplementary Table 4). This indicates that bilateral artefact burden is associated with increased dementia risk independently of ocular comorbidities. In multivariable regression, using all artefact variables demonstrated modest improvements in predicting incident dementia compared to the baseline (Table 3). Figure 2 shows the Kaplan–Meier curves where a higher cumulative incidence of cognitive decline in participants with artefacts in either eye and an increased risk of dementia conversion among participants with bilateral artefacts. Both curves highlight consistent patterns of greater vulnerability in those with artefact-prone scans.

**TABLE 4:** Associations between optical coherence tomography (OCT) artefacts and conversion from cognitive impairment, no dementia (CIND) to incident dementia (*n* = 222).

OCT artefact parameter	Model 1 HR (95% CI)	Model 1 *P*-value	Model 2 HR (95% CI)	Model 2 *P*-value
Poor scan quality	1.56 (0.75–3.26)	0.238	–	–
Types of artefacts				
Motion	1.08 (0.66–1.77)	0.761	–	–
Shadows	1.22 (0.73–2.04)	0.447	–	–
Low signal strength ( ≤ 5)	1.23 (0.64–2.37)	0.527	–	–
Off-center	0.98 (0.43–2.22)	0.959	–	–
Refractive shift	1.03 (0.36–2.98)	0.954	–	–
Out of boundary	1.23 (0.65–2.34)	0.528	–	–
Tilt	1.58 (0.55–4.48)	0.394	–	–
Presence of artefacts				
Artefacts in any eye	1.28 (0.65–2.51)	0.478	–	–
Artefacts in both eyes	**1.71 (1.05–2.78)**	**0.031**	**1.82 (1.10–3.02)**	**0.019**

CI, confidence interval; HR, hazard ratio. Artefacts in any eye = presence of ≥ 1 artefact in either eye. Artefacts in both eyes = presence of ≥ 1 artefact in each eye. *P*-values were obtained from Cox proportional hazards regression models. Model 1—Adjusted for age. Model 2—Adjusted for age, sex, education, and diabetes mellitus. Bold values denote statistical significance at *p* < 0.05.

## 4 Discussion

In this study, we demonstrated the potential prognostic value that OCT scan artefacts, typically regarded as indicators of poor image quality, carry for adverse cognitive outcomes. Across a large Asian memory clinic cohort followed for 5 years, the presence of any artefact was independently associated with significantly increased odds of cognitive decline, even after accounting for demographics, diabetes mellitus, and baseline cognition. In participants with CIND at baseline, artefacts in both eyes also predicted incidental progression to dementia. Collectively, these findings suggest that OCT artefact burden may represent a clinically relevant indicator of cognitive vulnerability.

These findings extend the potential of OCT utility beyond structural retinal measurements (Chua et al., 2022a; Eppenberger et al., 2024; Chua et al., 2025). Artefact-prone scans may capture acquisition difficulties related to underlying neurobiological vulnerability, such as impaired fixation, reduced attention, or subtle visuomotor dysfunction, which are commonly observed in prodromal or early dementia (Crawford et al., 2013; Crawford et al., 2015). This interpretation is consistent with our prior study (Foo et al., 2025), showing that poor image quality in retinal imaging modalities often correlates with the severity of cognitive impairment, rather than representing random noise. Additionally, while OCT artefact burden predicted subsequent cognitive decline and incident dementia in a time-to-event framework, incremental predictive value is relatively modest, and additional calibration for broader clinical utility is still necessary in future studies.

### 4.1 Comparisons with existing studies

Prior OCT studies have predominantly focused on structural retinal biomarkers, particularly thinning of the peripapillary RNFL and macular GCIPL, both consistently linked to cognitive decline (Eppenberger et al., 2024) and dementia (den Haan et al., 2017; Kwon et al., 2017; Shao et al., 2018; Chan et al., 2019; Jindahra et al., 2020; Noah et al., 2020). Our recent memory clinic-based study found that individuals with a thinner baseline macular GCIPL (≤79 μm) had a 38% increased risk of cognitive decline over 5 years, even after adjusting for vascular and demographic covariates, suggesting greater sensitivity of GCIPL over RNFL in early neurodegenerative changes (Eppenberger et al., 2024).

In contrast, scans with artefacts and poor quality are often excluded, rather than treated with prognostic value. Our prior work demonstrated that dementia is associated with substantially higher odds of poor-quality OCT scans, even after adjusting for demographics and comorbidity (Foo et al., 2025). To our knowledge, no study has systematically evaluated OCT artefact burden as a clinical marker. However, evidence from neuroimaging suggests that artefacts may themselves carry diagnostic information. In functional MRI, head motion artefacts have been shown to differ between participants with mild cognitive impairment, Alzheimer’s disease, and healthy controls, with motion parameters achieving modest discriminatory ability (area under curve 0.71–0.75) between groups (Haller et al., 2014). This study suggests that artefacts, traditionally dismissed as noise, may instead capture aspects of disease-related vulnerability. Artefact presence, particularly in both eyes, independently predicted subsequent cognitive decline and progression from CIND to dementia over 5 years, raising the possibility that artefact burden represents a novel and underexplored dimension of OCT-derived biomarkers.

### 4.2 Potential mechanisms/interpretation

The value of OCT artefacts as a predictor may be explained by the behavioural and brain changes that occur early in cognitive impairment. Difficulty in cooperating with imaging procedures, such as maintaining stable fixation and minimising head or eye movements, can result in poorer scan quality and may inadvertently serve as an early marker of cognitive dysfunction. Dementia and mild cognitive impairment are frequently associated with deficits in visuomotor control, impaired ocular motility, and reduced visual attention (Opwonya et al., 2023), all of which are crucial for acquiring high-quality OCT images. In our study, the most common artefact was motion artefacts (57%). Motion artefacts, characterised by horizontal discontinuity on the scan, arise primarily from participant’s difficulty in maintaining proper gaze fixation during the imaging process (Kastelan et al., 2025). Such fixation instability and attentional lapses align with established evidence of ocular motility dysfunctions and saccadic abnormalities in neurodegenerative disease contexts, specifically in Alzheimer’s disease (Javaid et al., 2016).

Peripapillary RNFL imaging using an optic nerve head (ONH) scan is a widely adopted OCT protocol in cognitive research, while macular scans, used to derive GCIPL thickness, represent another well-validated biomarker in Alzheimer’s disease and MCI (den Haan et al., 2017; Chan et al., 2019). Importantly, these two scan types differ in their attentional demands: macular imaging relies on a bright central fixation light that is generally easy to maintain, whereas ONH scans require subjects to fixate slightly off-centre on a peripheral target. This off-centre fixation may tax sustained attention and visuomotor coordination; difficulty maintaining it can produce artefacts and may reflect early attentional or executive dysfunction. Thus, beyond providing structural RNFL metrics, ONH scans may also indirectly index a participant’s capacity to engage with the imaging task itself, helping to explain the prognostic signal of artefact burden observed in our study.

Taken together, our findings suggest that artefact-prone OCT scans may not simply represent technical error, but could also capture subtle visuomotor, attentional, or task-engagement difficulties that may accompany early neurocognitive decline. OCT artefact burden should therefore be interpreted as an acquisition-related marker potentially associated with cognitive decline or dementia, rather than as disease-specific pathological process.

### 4.3 Distinction between predictors of general cognitive decline and conversion from CIND to dementia

In this study, we evaluated two related but distinct outcomes: firstly, cognitive decline in the entire cohort and secondly, progression from CIND to dementia. In the overall cohort (*n* = 507), the presence of any OCT artefact at baseline was independently associated with a 37% higher risk of cognitive decline over 5 years (HR = 1.37, 95% CI 1.12–1.68, *P* = 0.003), even after adjusting for age, sex, education, diabetes, and baseline diagnosis. Although motion and off-center artefacts were more frequent among decliners, overall artefact burden proved to be a stronger and more consistent predictor than any individual artefact subtype.

In contrast, among participants with CIND at baseline (*n* = 222), bilateral artefacts were associated with an 82% higher likelihood of conversion to dementia. Bilateral involvement may indicate more widespread deficits in visuomotor control and attentional regulation, reflecting more extensive cortical dysfunction during the prodromal phase (Tippett and Sergio, 2006; Verheij et al., 2012). Thus, overall artefact burden serves as a marker of vulnerability to cognitive decline across the spectrum, whereas bilateral artefacts provide specific prognostic information for identifying individuals at intermediate disease stages who are at heightened risk of progression to dementia.

### 4.4 Clinical implications

Our findings suggest that OCT artefact burden should not necessarily be dismissed as simple image noise (Han and Jaffe, 2010; Bazvand and Ghassemi, 2020; Holmen et al., 2020), but may represent a low-cost and readily obtainable indicator of cognitive vulnerability. Because artefact metrics are routinely graded during OCT quality control, they represent a “free” by-product of imaging that could enhance case detection in eye clinic settings. For example, the presence of artefacts in both eyes may help identify individuals who could benefit from closer cognitive assessment or monitoring.

Importantly, our results highlight different prognostic utilities: artefacts in either eye predict risk of cognitive decline across the spectrum, whereas bilateral artefacts specifically identify individuals with CIND at greater risk of conversion to dementia. Integrating these simple metrics with established structural OCT measures (Chua et al., 2022a; Eppenberger et al., 2024; Chua et al., 2025) could support earlier identification of at-risk individuals. Specifically, future studies should examine whether incorporating artefact metrics alongside established anatomical biomarkers such as RNFL or GCIPL thickness improves predictive model performance and hence clinical utility.

A further direction would also be to clarify the temporal sequence of retinal biomarker abnormalities during progression toward dementia. Multimodal longitudinal studies could determine whether acquisition-related functional difficulty, as reflected by artefact burden, emerges earlier than measurable structural thinning, or whether these changes evolve in parallel.

Optical coherence tomography angiography (OCTA), while providing vascular biomarkers linked to Alzheimer’s disease and mild cognitive impairment (Chua et al., 2020; Chua et al., 2024), is also highly susceptible to artefacts. Because OCTA is generally more artefact-prone than structural OCT, its artefact burden may even prove more sensitive as a marker of cognitive or visuomotor impairment. However, this possibility remains largely unexplored.

### 4.5 Strengths

Our study has several strengths. First, it leverages a prospective, longitudinal design with up to 5 years of follow-up in a well-characterised memory clinic cohort. Second, cognitive outcomes were rigorously assessed using a comprehensive, standardised neuropsychological battery, ensuring clinical relevance and consistency. Third, the use of systematic artefact grading blinded to cognitive status, with multivariable adjustment for demographic, ocular, and clinical factors strengthened the evidence for its independent prognostic value. Finally, by evaluating both overall cognitive decline in the full cohort and progression from CIND to dementia in a targeted subgroup, our study shows that OCT artefacts provide valuable information across different stages of cognitive impairment, from early risk to advanced disease progression.

### 4.6 Limitations

Our study has several limitations. First, artefact grading was performed manually, which may introduce subjectivity despite standardised criteria. Automated artefact detection using artificial intelligence could enhance reproducibility in future studies. Second, while we adjusted for demographics, comorbidities, and baseline cognition, residual confounding from unmeasured factors such as attention deficits or motor dysfunction cannot be excluded. Third, our cohort was derived from memory clinics and predominantly Asian, which may limit generalisability to community populations or other ethnic groups. Fourth, our cohort included participants with common ocular co-morbidities such as age-related macular degeneration, diabetic retinopathy, glaucoma (Chua et al., 2022b), and their prevalence and distribution were similar between those with and without cognitive decline. Because our focus was artefact burden, we included eyes with these conditions as long as imaging was technically feasible. The findings should therefore be applicable to similar memory-clinic cohorts but may not automatically extrapolate to populations with a very different ocular disease profile.

A further limitation is the non-specific nature of OCT artefacts. Artefacts may arise not only from dementia-related dysfunction, but also from transient or incidental factors during image acquisition, including blinking, dry eyes, or fatigue. As such, the observed associations should not be interpreted as evidence that OCT artefacts are specific markers of dementia-related impairment. In this study, image acquisition was performed by trained technicians using standardised protocols, with repeat scans obtained when scan quality was suboptimal. This practice may reduce the influence of purely random or transient events on artefact detection. Nevertheless, OCT artefacts remain a non-specific measure of imaging difficulty, and further studies are needed to determine the extent to which they reflect cognitive, visuomotor, behavioural, or other acquisition-related factors.

## 5 Conclusion

OCT artefacts, long considered as impediments to imaging, may instead provide valuable insight into underlying neurodegenerative processes. The presence of artefacts was independently associated with cognitive decline and incident dementia. These findings highlight the potential of artefact assessment as an opportunistic, non-invasive screening tool for dementia risk stratification. Although longitudinal associations were observed, mechanistic links between OCT artefacts and neurodegeneration remain speculative and require further validation.

## Data Availability

The raw data supporting the conclusions of this article will be made available by the authors, without undue reservation.
