# Supplementary material for: Optical coherence tomography artefact burden predicts cognitive decline and incident dementia
Source: Front Aging Neurosci. 2026 May 15;18:1802753. doi: 10.3389/fnagi.2026.1802753 (PMC13219028; doi:10.3389/fnagi.2026.1802753)
Supplement: Supplementary file 1 [file Data_Sheet_1.docx]

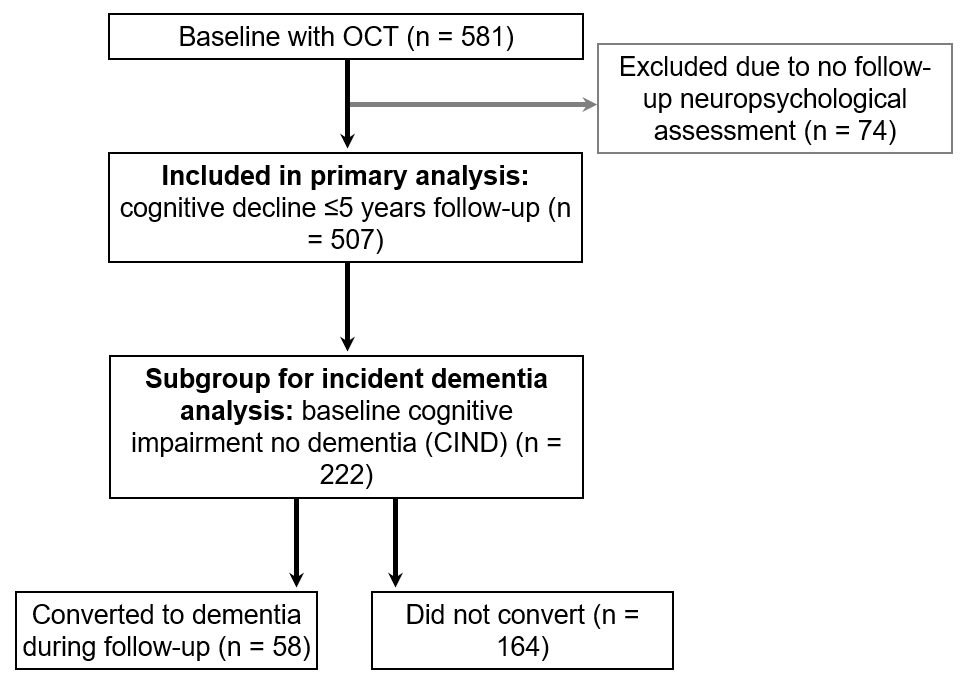


**Supplementary Figure 1: Flow chart of the study.** Among 581 participants with baseline optical coherence tomography (OCT) imaging, 74 were excluded due to missing follow-up neuropsychological data, leaving 507 participants for the primary analysis of cognitive decline. The incident dementia analysis focused on the subgroup with baseline CIND (n = 222), of whom 58 converted to dementia over follow-up (164 did not convert).


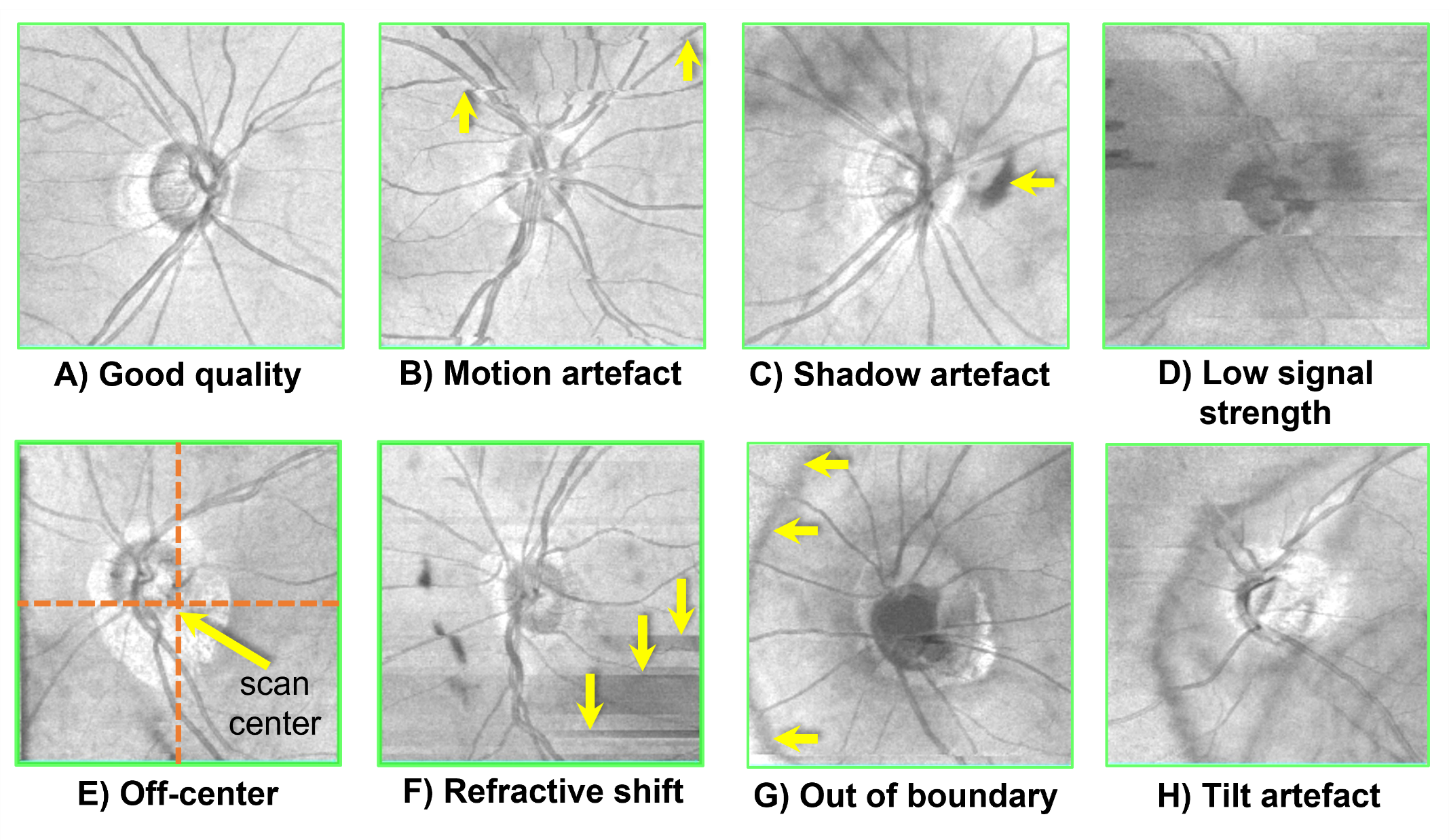


**Supplementary Figure 2.** Representative examples of artefact types encountered during optical coherence tomography (OCT) imaging. (A) Good-quality scan without visible artefacts. (B) Motion artefact, characterised by horizontal discontinuities of retinal vessels (arrows). (C) Shadow artefact, showing localized signal attenuation from vitreous opacity or media opacity (arrow). (D) Low signal strength, with diffuse reduction in image contrast and retinal layer visibility. (E) Off-center scan, indicated by displacement of the optic disc relative to the scan center (orange dashed lines and arrow). (F) Refractive shift artefact, characterised by banding or intensity variation between adjacent scan regions (arrows). (G) Out-of-boundary artefact, where part of the retinal image is cropped at the scan margins (arrows). (H) Tilt artefact, showing uneven illumination or defocus due to poor alignment.

| **Supplementary Table 1. Comparison of optical coherence tomography artefacts across diagnostic groups** | | | | |
| --- | --- | --- | --- | --- |
| **Characteristics** | **Dementia** | **Cognitive impairment no dementia** | **No cognitive impairment** | **P value** |
|  |  |  |  |  |
| **Number of subjects** | 174 | 222 | 111 |  |
| **Poor scan quality** | 52 (30%) | 19 (9%) | 9 (8%) | **<0.001** |
| **Types of artefacts** |  |  |  |  |
| Motion | 106 (61%) | 106 (48%) | 48 (43%) | **0.005** |
| Shadows | 84 (48%) | 114 (51%) | 52 (47%) | 0.700 |
| Low signal strength (≤5) | 21 (12%) | 27 (12%) | 5 (5%) | 0.068 |
| Off-center | 25 (14%) | 23 (10%) | 5 (5%) | **0.030** |
| Refractive shift | 19 (11%) | 15 (7%) | 5 (5%) | 0.110 |
| Out of boundary | 11 (6%) | 26 (12%) | 11 (10%) | 0.188 |
| Tilt | 5 (3%) | 5 (2%) | 4 (4%) | 0.773 |
| **Presence of artefacts** |  |  |  |  |
| Any artefacts | 136 (78%) | 166 (75%) | 73 (66%) | 0.063 |
| Better-eye artefacts | 72 (41%) | 80 (36%) | 34 (31%) | 0.179 |
| P-values were obtained using chi-squared test and Fisher’s exact test was applied when expected cell counts were <5. | | | | |

**Supplementary Table 2. Associations between optical coherence tomography artefacts at baseline and risk of cognitive decline, compensated for presence of ocular disease**

|  | **Model 1** | | **Model 2** | |
| --- | --- | --- | --- | --- |
| **Subjects (n = 468)** | **HR (95% CI)** | **P value** | **HR (95% CI)** | **P value** |
| **Poor scan quality** | 1.30 (0.96-1.77) | 0.091 | 1.04 (0.77-1.42) | 0.778 |
| **Types of artefacts** |  |  |  |  |
| Motion | **1.36 (1.09-1.70)** | **0.006** | 1.19 (0.95-1.49) | 0.123 |
| Shadows | 1.10 (0.89-1.38) | 0.373 | 1.13 (0.91-1.40) | 0.261 |
| Low signal strength (≤5) | 0.79 (0.53-1.16) | 0.230 | 0.72 (0.49-1.06) | 0.094 |
| Off-center | 1.30 (0.93-1.81) | 0.126 | 1.10 (0.77-1.58) | 0.603 |
| Refractive shift | 1.23 (0.83-1.81) | 0.309 | 1.11 (0.76-1.62) | 0.599 |
| Out of boundary | 1.17 (0.81-1.68) | 0.403 | 1.35 (0.93-1.96) | 0.109 |
| Tilt | 0.96 (0.48-1.95) | 0.913 | 1.03 (0.52-2.03) | 0.941 |
| **Presence of artefacts** |  |  |  |  |
| Artefacts in any eye | **1.51 (1.15-2.00)** | **0.003** | **1.38 (1.06-1.79)** | **0.017** |
| Artefacts in both eyes | 1.24 (1.00-1.55) | 0.052 | 1.15 (0.93-1.43) | 0.201 |

CI, confidence interval; HR, hazard ratio

Artefacts in any eye = presence of ≥1 artefact in either eye. Artefacts in both eyes = presence of ≥1 artefact in each eye.

P-values were obtained from Cox proportional hazards regression models.

Model 1 - Adjusted for age, and presence of ocular disease.

Model 2 - Adjusted for age, sex, education, diabetes mellitus, baseline diagnosis, and presence of ocular disease.

Bold values denote statistical significance at p < 0.05.

| **Supplementary Table 3. Comparison of demographic and clinical characteristics among cognitive impairment, no dementia (CIND) subjects who converted to dementia and those who did not convert to dementia over a 5-year follow-up period** | | | |
| --- | --- | --- | --- |
| **Characteristics** | **Converted to dementia** | **No conversion** | **P value** |
|  |  |  |  |
| **Number of subjects** | 58 | 164 |  |
| **Age (years)** | 77 (7) | 74 (10) | **0.007** |
| **Sex, male** | 24 (41%) | 79 (48%) | 0.373 |
| **Education (years)** | 8 (8) | 8 (6) | 0.936 |
| **Diabetes, yes** | 19 (33%) | 54 (33%) | 0.981 |
| **Hypertension, yes** | 37 (64%) | 103 (63%) | 0.935 |
| **Blood pressure** |  |  |  |
| Systolic blood pressure (mmHg) | 140 (24) | 142 (22) | 0.569 |
| Diastolic blood pressure (mmHg) | 71 (14) | 76 (12) | **0.036** |
| **Cognitive tests** |  |  |  |
| MMSE total score | 22 (6) | 25 (4) | **<0.001** |
|  |  |  |  |
| **Ocular characteristics** |  |  |  |
| Spherical equivalent (diopters) | -0.50 (1.5) | -0.38 (2) | 0.262 |
| Signal strength of scan (0 poor to 10 good) | 7 (3) | 8 (2) | 0.110 |
| Average RNFL thickness (µm) | 87 (16) | 89 (16) | 0.124 |
| Ocular disease in any eye | 14 (25%) | 65 (41%) | **0.029** |
|  |  |  |  |
| **Scan quality** |  |  |  |
| Good scan quality in either eye | 51 (88%) | 152 (93%) | 0.266 |
| Poor scan quality in both eyes | 7 (12%) | 12 (7%) |  |
| **Artefacts** |  |  |  |
| Motion | 28 (48%) | 78 (48%) | 0.925 |
| Shadows | 35 (60%) | 79 (48%) | 0.111 |
| Low signal strength (≤5) | 10 (17%) | 17 (10%) | 0.169 |
| Off-center | 6 (10%) | 17 (10%) | 0.996 |
| Refractive shift | 4 (7%) | 11 (7%) | 0.961 |
| Out of boundary | 9 (16%) | 17 (10%) | 0.294 |
| Tilt | 2 (3%) | 3 (2%) | 0.475 |
| Any artefacts | 47 (81%) | 119 (73%) | 0.202 |
| Better-eye artefacts | 29 (50%) | 51 (31%) | **0.010** |
| Data provided in median (IQR) or number (%). | | | |
| MMSE, Mini-Mental State Examination | | | |
| P-values were obtained using Kruskal-Wallis test for continuous variables and the chi-squared test for categorical variables; Fisher’s exact test was applied when expected cell counts were <5. | | | |
| Bold values denote statistical significance at the p < 0.05 level. | | | |

**Supplementary Table 4. Associations between optical coherence tomography (OCT) artefacts and conversion from cognitive impairment, no dementia (CIND) to incident dementia, compensated for presence of ocular disease**

|  | **Model 1** | | **Model 2** | |
| --- | --- | --- | --- | --- |
| **Subjects (n = 213)** | **HR (95% CI)** | **P value** | **HR (95% CI)** | **P value** |
| **Poor scan quality** | 1.80 (0.91-3.59) | 0.093 | 1.96 (0.95-4.04) | 0.069 |
| **Types of artefacts** |  |  |  |  |
| Motion | 1.19 (0.71-1.97) | 0.510 | 1.31 (0.77-2.23) | 0.328 |
| Shadows | 1.23 (0.73-2.07) | 0.432 | 1.28 (0.76-2.17) | 0.359 |
| Low signal strength (≤5) | 1.52 (0.77-2.99) | 0.225 | 1.59 (0.78-3.24) | 0.204 |
| Off-center | 0.96 (0.41-2.22) | 0.923 | 1.04 (0.44-2.47) | 0.929 |
| Refractive shift | 0.70 (0.21-2.37) | 0.569 | 0.59 (0.16-2.23) | 0.436 |
| Out of boundary | 1.23 (0.64-2.39) | 0.533 | 1.24 (0.63-2.47) | 0.532 |
| Tilt | 1.59 (0.51-5.01) | 0.426 | 1.44 (0.40-5.13) | 0.574 |
| **Presence of artefacts** |  |  |  |  |
| Artefacts in any eye | 1.30 (0.66-2.57) | 0.446 | 1.40 (0.69-2.83) | 0.350 |
| Artefacts in both eyes | 1.61 (0.98-2.65) | 0.061 | **1.70 (1.02-2.83)** | **0.040** |

CI, confidence interval; HR, hazard ratio

Artefacts in any eye = presence of ≥1 artefact in either eye. Artefacts in both eyes = presence of ≥1 artefact in each eye.

P-values were obtained from Cox proportional hazards regression models.

Model 1 - Adjusted for age, and presence of ocular disease.

Model 2 - Adjusted for age, sex, education, diabetes mellitus, baseline diagnosis, and presence of ocular disease.

Bold values denote statistical significance at p < 0.05.
